# Gibberellins Promote Brassinosteroids Action and Both Increase Heterosis for Plant Height in Maize (*Zea mays* L.)

**DOI:** 10.3389/fpls.2017.01039

**Published:** 2017-06-20

**Authors:** Songlin Hu, Cuiling Wang, Darlene L. Sanchez, Alexander E. Lipka, Peng Liu, Yanhai Yin, Michael Blanco, Thomas Lübberstedt

**Affiliations:** ^1^Department of Agronomy, Iowa State University, AmesIA, United States; ^2^Department of Agronomy, Henan University of Science and TechnologyLuoyang, China; ^3^Department of Crop Sciences, University of Illinois at Urbana–Champaign, ChampaignIL, United States; ^4^Department of Statistics, Iowa State University, AmesIA, United States; ^5^Department of Genetics, Development and Cell biology, Iowa State University, AmesIA, United States; ^6^Plant Introduction Research Unit, Department of Agronomy, United States Department of Agriculture – Agricultural Research Service, Iowa State University, AmesIA, United States

**Keywords:** brassinosteroid, gibberellin, plant height, genome-wide association study, heterosis

## Abstract

Brassinosteroids (BRs) and Gibberellins (GAs) are two classes of plant hormones affecting plant height (PHT). Thus, manipulation of BR and GA levels or signaling enables optimization of crop grain and biomass yields. We established backcross (BC) families, selected for increased PHT, in two elite maize inbred backgrounds. Various exotic accessions used in the germplasm enhancement in maize project served as donors. BC1-derived doubled haploid lines in the same two elite maize inbred backgrounds established without selection for plant height were included for comparison. We conducted genome-wide association studies to explore the genetic control of PHT by BR and GA. In addition, we used BR and GA inhibitors to compare the relationship between PHT, BR, and GA in inbred lines and heterozygotes from a physiological and biological perspective. A total of 73 genomic loci were discovered to be associated with PHT, with seven co-localized with GA, and two co-localized with BR candidate genes. PHT determined in field trials was significantly correlated with seedling stage BR and GA inhibitor responses. However, this observation was only true for maize heterozygotes, not for inbred lines. Path analysis results suggest that heterozygosity increases GA levels, which in turn promote BR levels. Thus, at least part of heterosis for PHT in maize can be explained by increased GA and BR levels, and seedling stage hormone inhibitor response is promising to predict heterosis for PHT.

## Introduction

Increasing demand for biomass production led to a paradigm shift regarding plant height (PHT) from dwarfs to giants (Fernandez et al., 2009). Tall maize varieties are more desirable, if maize is used as dual purpose or biomass crop. For dual-purpose maize, increased stover biomass adds value: grain is harvested for food or feed, stover for bioenergy conversion. While PHT increasing alleles contribute to increasing biofuel production, they also increase the risk of lodging. To overcome this negative side effect, breeders can either produce maize varieties with increased lignin level or a stronger root system to stabilize plants (Fernandez et al., 2009). Increased lignin levels are not desirable for biochemical, but favorable for thermochemical conversion of biomass (Mendu et al., 2011).

Plant height is an important agronomic trait in modern maize and more generally cereal breeding programs, and has been manipulated during maize domestication as it shows significant correlations with different agronomic traits such as grain yield (Teng et al., 2013; Abdel-Ghani et al., 2016). For grain maize production, breeders prefer a short stature, as high yielding maize varieties need to be lodging-tolerant under high nitrogen levels and high density planting conditions. Breeders use semi-dwarf genes to moderately decrease PHT in cereals, such as the green revolution genes *sd-1* in rice (Sasaki et al., 2002) and *Rht* in wheat (Peng et al., 1999). In maize, several maize dwarf genes have been well-characterized, but are not intentionally used in breeding programs due to their adverse impact on grain yield, such as *dwarf3* (Winkler and Helentjaris, 1995), *dwarf8 and dwarf9* (Thornsberry et al., 2001; Lawit et al., 2010), *nana plant1* (Hartwig et al., 2011), and *brd1* (Makarevitch et al., 2012). All these genes cause defects in either the brassinosteroid (BR) or the gibberellin (GA) pathway, stressing the importance of these two plant hormones in the control of PHT. It was reported that BR and GA have the most direct effects on PHT without major negative pleiotropic effects as compared to other plant hormones (Fernandez et al., 2009).

Brassinosteroids are steroid hormones found throughout the plant kingdom, similar to animal steroid hormones. They promote cell growth, even if at low concentrations, by regulating cell division and elongation (Clouse, 1996). The biosynthesis pathway for brassinolide (BL), the most active BR, is well-established by characterization of BR-deficient mutants in model species such as Arabidopsis, pea (*Pisum sativum*), and tomato (*Solanum lycopersicum*). Enzymes involved in BL biosynthesis include DWARF7 (DWF7), DIMINUTO (DIM1), FAD dependent oxidase, DEETIOLATED2 (DET2) steroid 5a-reductase and several cytochrome p450 monooxygenases, such as DWF4, CONSTITUTIVE PHOTOMORPHOGENESIS AND DWARFISM (CPD), and DWF (Fernandez et al., 2009). Signal transduction (Clouse, 2011) initiates with binding of BR to BRASSINOSTEROID INSENSITIVE 1 (BRI1), a plasma membrane-localized leucine rich repeat (LRR) receptor kinase. In the absence of BR, the negative regulator (BRI1 KINASE INHIBITOR 1) BKI1 binds and inhibits BRI1 function (Jaillais and Chory, 2010). Transcription factors (bri1-EMS-suppressor 1) BES1/(bri1 ETHLYMETHANESULFONATE SUPPRESSOR1 and BRASSINAZOLE RESISTANT1) BZR1 are phosphorylated by (BRASSINOSTEROID INSENSITIVE2) BIN2 and are inhibited by several mechanisms (Choe et al., 2002). Binding of BR to BRI1 leads to release of BKI1, which interacts with 14-3-3 and thus promotes nuclear accumulation of BES1 (Wang et al., 2011). Association of BRI1 with co-receptor (BRASSINOSTEROID INSENSITIVE 1) BAK1 activates BRI1 kinase activity (Gou et al., 2012). Activated BRI1 signals through (BR-Signaling Kinases) BSK1 and (Constitutive Differential Growth1) CDG1 kinases as well as (BRI1-Supressor1) BSU1 phosphatase to inhibit BIN2 kinase activity (Kim et al., 2011). The inhibition of BIN2 and action of (protein phosphatase 2A) PP2A phosphatase allow accumulation of BES1/BZR1 in the nucleus, which regulates gene expression in combination with other transcription regulators (Tang et al., 2010).

Gibberellins are cyclic diterpene compounds that promote stem elongation, and mutants in GA synthesis or signaling show dwarf phenotypes. GA synthesis begins with *trans*-geranylgeranyl diphosphate and requires six enzymatic steps for the formation of bioactive GA1 or GA4 (Yamaguchi, 2008). A deficiency in any step leading to the production of bioactive GAs causes dwarfism. GA homeostasis is maintained through a balance of anabolic and catabolic activities. Four rice genes are primarily responsible for this regulation through a feedback transcriptional control mechanism: (GA 20-oxidase) GA20ox, GA3ox, GA2ox, and (epioxidation catalyzed by ELONGATEDUPPER INTERNODES1) EUI1 (Appleford et al., 2007). All of them are proven targets for manipulating GA levels. For example, the recessive tall rice mutant elongated uppermost internode (eui) (Rutger and Carnahan, 1981) exhibits a rapid and enhanced elongation of internodes, particularly the uppermost internode during the heading stage. It has been shown that EUI controls bioactive GA levels to modulate internode elongation in a tissue- and developmental stage–specific manner (Zhu et al., 2006). Central to GA signaling are DELLA proteins, which are negative regulators that repress GA-induced gene transcription in the absence of GA signaling (Zentella et al., 2007). GA signaling induces gene expression by targeting the DELLA proteins for degradation (GIBBERELLIN INSENSITIVE DWARF1) GID1 and GID2 are positive regulators of GA signaling and (SLENDER RICE1) SLR1 is a DELLA protein and is a negative regulator (Sasaki et al., 2003) of GA signaling. As with metabolism, both positive and negative regulators of GA signaling have the demonstrated potential for dramatic effects on PHT.

Plant hormone inhibitors are powerful tools for elucidating plant hormone functions during plant development. For example, BR inhibitor Propiconazole (Pcz) has successfully been employed for studying BR control of sex determination and PHT in maize (Hartwig et al., 2012). GA inhibitor Uniconazole (Ucz) was used to explore root growth and nitrogen transfer in soybean (Yan et al., 2013). Instead of using hormone pathway mutants, plant hormone inhibitors can phenocopy hormone-deficient mutants in crops, with the advantage that deficiency levels of hormones can be controlled. Moreover, plant hormone inhibitors can help with identification and characterization of hormone deficient mutants without prior knowledge of the mutant phenotype (Hartwig et al., 2012). In maize, Pcz and Ucz are two popular plant hormone inhibitors of BR and GA, respectively, due to their easy accessibility and low costs (Rademacher, 2000; Hartwig et al., 2012). Application of Pcz and Ucz reduce mesocotyl elongation, and genotypes with elevated BR or GA level are more tolerant to Pcz or Ucz, resulting in alleviated reduction of mesocotyl length (Hartwig et al., 2012). Therefore, Pcz and Ucz can be employed to explore the relationship between morphological traits, BR and GA activities in maize.

In this study, two sets of backcross (BC) libraries derived by introgression of a diverse set of tropical maize accessions into inbred lines, representing two major maize heterotic groups (Iowa Stiff Stalk and Non-Stiff Stalk), were evaluated for PHT. Moreover, we applied BR and GA inhibitors Pcz and Ucz at seedling stage to compare BR and GA inhibitor responses between tall and short maize BC families within each library, and between the tallest and shortest individuals within BC families. In addition, two sets of doubled haploid (DH) libraries derived from the same parents producing the two BC libraries were tested for PHT and BR/GA activities as a comparison. Our objectives were (1) to evaluate the PHT performance of the two BC libraries and the two DH libraries; (2) to conduct a genome-wide association study (GWAS) to investigate the genomic regions associated with BR, GA, and PHT in these BC families; and (3) to apply Pcz and Ucz treatments to these two BC libraries and two DH libraries to investigate the relationship between BR, GA, and PHT in both inbred lines (the two DH libraries) and heterozygotes (the two BC libraries).

## Materials and Methods

### Plant Materials

Two libraries (refer to an enriched genetic diversity) of phenotypic-selected introgression families (PIFs): PIFB47 (PHB47 as recurrent parent) and PIFZ51 (PHZ51 as recurrent parent) were used in this study. PHB47 and PHZ51 are two elite expired PVP (Plant Variety Protection) inbred lines which belong to Iowa Stiff Stalk and Non-Stiff Stalk heterotic groups, respectively. Donor parents were tropical or sub-tropical accessions from the germplasm enhancement of maize (GEM) project (Salhuana and Pollak, 2006), of which 42 different accessions were used for PIFB47 and 46 for PIFZ51, with five accessions being used for both PIFB47 and PIFZ51 (**Supplementary Table S1**). The process of constructing PIF libraries was described in a previous study (Abdel-Ghani et al., 2016). Briefly, in each backcross (BC) generation, phenotypic selection for PHT (selection for tallness) and flowering time (synchrony with recurrent parent) was carried out to accumulate PHT increasing alleles from donor parents into elite maize background (PHB47 and PHZ51) and to minimize confounding effects between flowering time and PHT. As a result, 75 and 71 PIFs were produced for PIFB47 and PIFZ51, respectively (**Supplementary Table S1**). In addition, two doubled haploid libraries (DHB47 and DHZ51) were used as unselected groups (without any phenotype selection) for comparison, including 103 and 66 BGEM (DH lines from GEM project) lines, respectively. The method used to create the BGEM lines was described in a previous study (Brenner et al., 2012). Briefly, donor parents from GEM project were used for producing BGEM lines as for PIFs, and same recurrent parents (PHB47 and PHZ51) were used for backcrossing. Different from PIF development, BGEM lines were produced (induced and doubled) from BC_1_ individuals, and there was no phenotypic selection during the backcross process. The hybrid of PHB47 × PHZ51 was included in field experiments for comparison.

### Field Experiments

#### Experiment 1: PHT and Flowering Time Characterization of PIFs

PIFs together with their recurrent parents were evaluated for PHT across 3 years (2013, 2014, 2015), with one location in 2013 (Plant Introduction Station: PSI, Ames, IA, United States), three locations in 2014 [Agronomy farm (AG), Ames, IA, United States PSI, and Neely-Kinyon Memorial Research and Demonstration Farm (NK) – Greenfield, IA, United States] and one location in 2015 (AG) with a randomized complete block design (RCBD) within each location (two blocks per location). All locations were under standard farm management practices (tillage, nutrient management, etc.), with 15 plants per plot and 0.75 m between plots. PIFs were compared with their recurrent parents and hybrid of PHB47 × PHZ51 for PHT and flowering time. Two weeks after tasseling, a representative plant with median PHT (MPHT) was selected per plot, and PHT was recorded from ground to tassel tip. Across years and locations, multiple data points for each PIF were collected. In 2014, the shortest (SPHT) and the tallest individual (TPHT) within each PIF were also measured. In 2014 and 2015, flowering time traits – days to tasseling (DTT), days to silking (DTS), and anthesis-silking interval (ASI) were recorded for each plot, when at least 50% of the plants shed pollen or showed silks.

#### Experiment 2: PHT and Flowering Time Characterization of BGEM Lines

DHB47, DHZ51 together with their recurrent parent were characterized for PHT and flowering time in 2014 and 2015 with a RCBD design with two replications at AG. In each plot, one representative individual grown in the middle of the plot was used to measure PHT, and flowering time was measured in the same way as for PIFs.

#### Experiment 3: Phenotypic and Genotypic Characterization for the Two Tallest Individuals within Each PIF

In 2014 at PSI, leaf samples of the two tallest plants from each PIF together with six PHB47 and six PHZ51 individuals (304 individuals in total) were collected after tasseling for genotyping. PHT, ear height (EHT), node number (NNode), leaf angle of the first leaf below the flag leaf (LA) and tassel length (TL) were measured for each genotyped individual. EHT was measured from the ground to the primary ear node. NNode was scored as the number of nodes between the top brace root node and the flag leaf excluding the variation in brace root nodes and subterranean nodes. LA was measured as the angle between horizontal position and the midrib of the first leaf below the flag leaf. TL was measured from the non-branching node present below the lowermost primary branch to the tip of central spike. In 2015, the tallest and shortest individual from each PIF was selected to produce backcross seed for hormone inhibitor response comparison.

### Hormone Inhibitor Test Experiments

All PIFs and BGEM lines were evaluated for responses to BR and GA inhibitors in three independent experiments (BR, GA, and water). All entries were treated with 80 μM BR inhibitor Pcz, 80 μM GA inhibitor Ucz and water (mock treatment), with three replications per experiment in a completely randomized design (CRD). Each treatment was applied to 12 kernels from each PIF, and 6 kernels from each BGEM line for each replication within each experiment. Seed was soaked for 24 h, then transferred to paper rolls (Kumar et al., 2012) containing the same soaking solution (i.e., water, Pcz, or Ucz). Rolls were placed in buckets with a cover in a growth chamber without light at 25°C. After 8 days, seedlings were removed from the growth chamber and mesocotyl length was measured from the root-shoot transition zone to the first node for each seedling (Hartwig et al., 2012) as an indicator for hormone inhibitor response. The average mesocotyl length for the 12 (or 6) seedlings under Pcz, Ucz, and water treatment was recorded as MP (mesocotyl length with Pcz treatment), MU (mesocotyl length with Ucz treatment), and MW (mesocotyl length with water treatment), respectively. BR inhibitor response was defined as MP/MW, and GA inhibitor response as MU/MW according to a previous protocol (Hartwig et al., 2012; Huang et al., 2013), as shown in Supplementary Figure S1. Seed harvested from the tallest and the shortest individual within each PIF in 2015 was tested using the same protocol described above in three independent experiments.

### DNA Extraction and Genotyping

DNA was extracted using Qiagen BioSprint96 at the Genomic Technology Facility at Iowa State University. Samples were genotyped with the MaizeSNP3K chip (3072 SNPs across the whole genome), which is a subset of the Illumina MaizeSNP50 BeadChip (Ganal et al., 2011). Genotyping was executed using the Illumina GoldenGate SNP genotyping platform (Fan et al., 2006) at the National Maize Improvement Center of China (China Agricultural University). DNA from all BGEM lines was extracted using 1–10 kernels per line, and genotyped with 8523 (chip-based) SNPs across the genome, in which 7739 polymorphic markers were used in the analysis, with 920 markers overlapping with the MaizeSNP3K subset across the genome. Both DNA extraction and SNP chip genotyping of the BGEM lines were done at KWS SAAT SE (company in Germany).

### Marker Quality Control

The 12 genotyped recurrent parent individuals (six PHB47 and six PHZ51) were analyzed for marker quality control: SNP markers missing across all RP individuals or showing heterozygous state across at least three (out of six) recurrent parent individuals were excluded, as residual heterozygosity is random and unlikely to frequently occur at the same genomic position within a fixed inbred line. Subsequently, all PIF genotypes were jointly analyzed. SNP markers with >5% missing data were discarded. For missing SNP markers, the LD *k*-nearest neighbor algorithm (LD KNNi imputation) was used for imputation using TASSEL5.2.15 (Money et al., 2015).

### Phenotype and Genotype Statistical Analysis

Phenotypic data was analyzed using SAS 9.3 software (SAS Institute). For each evaluated trait, variance component estimates were obtained from a mixed linear model (MLM) fitted across all environments in SAS PROC MIXDED. Variance components (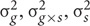) were estimated where 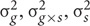 corresponds to genotypic variance, genotype by environmental interaction variance, and error variance respectively. Entry mean-based heritability was calculated from variance components estimates as h^2^ = 
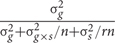
 where r is the number of replications within each location, and n is the number of locations (Holland et al., 2003). Comparisons between PIFs and recurrent parents or between DH lines and recurrent parents were done using Fisher’s least significant difference (LSD) with a significance level of 0.05. Pearson’s correlation coefficients were used to assess the relationship between different traits with the SAS PROC CORR function. Linear regression analysis was performed using the SAS PROC GLM function. Graphs were obtained using ggplot2 in R (Wickham, 2009).

### Genome-Wide Association Study

We conducted GWAS for all measured field traits and BR/GA inhibitor responses of the PIFs (292 genotypes) with 2930 polymorphic genome-wide SNPs (minor allele frequency > 5%). Population structure was estimated using Structure 2.3.4 software (Pritchard et al., 2000). The parameter settings included a burn-in length of 50,000 followed by 50,000 iterations of setting K (clusters – number of subpopulations) from 1 to 10, with five replications for each K (Pace et al., 2014). The most probable K value was picked by plotting each K (x-axis) with its estimated Ln probability of data (y-axis). The K value was selected when the estimated Ln probability of data reached a plateau (Pritchard et al., 2000).

To balance false positives and false negatives, we used three models for GWAS analysis: (1) a generalized linear model (GLM) + Q, with the covariates Q from STRUCTURE included in the model as fixed effects to account for population structure; (2) a MLM (Yu et al., 2006) with population structure and kinship as covariates; and (3) FarmCPU (Fixed and random model Circulating Probability Unification) with kinship and population structure as covariates, but with additional algorithms solving the confounding problems between testing markers and covariates (Liu et al., 2016). GLM was conducted with the software program TASSEL 5.0 (Bradbury et al., 2007). MLM was used as implemented in GAPIT (Genome Association and Prediction Integrated Tool-R package) (Lipka et al., 2012). FarmCPU was applied with R package FarmCPU (Liu et al., 2016). Statistical program simpleM implemented in R was used to account for multiple testing (Johnson et al., 2010). The threshold level was based on an effective number of independent tests m, and m was used in a similar way as the Bonferroni correction method (Pace et al., 2014). In this study, for a family-wise error rate of 0.05 the threshold for significant trait-marker associations was set as 4.44 × 10^-5^ (multiple testing threshold level).

### Comparison with Published PHT QTL Regions

SNP markers associated with PHT were compared with previously published PHT quantitative trait loci (QTL). The dataset for comparison is based on a maize PHT QTL meta-analysis aimed at identifying the most likely position of PHT QTL that are consistently found across studies. This dataset summarized published PHT QTL into 40 hot spots across the maize genome based on the maize B73 RefGen_v2 physical map (Wang et al., 2006). PHT associated SNP markers from this study were compared with these hot spots to study co-localizations.

### BR and GA Candidate Genes

The information about the genes involved in BR and GA biosynthesis and signaling pathway was collected from Arabidopsis and rice (model species) and listed in **Supplementary Table S2**. The protein sequence of these genes was obtained from the National Center for Biotechnology Information (NCBI) databases, and was used to identify orthologous genes in maize through BLASTP in Gramene (Altschul et al., 1997). Based on BLASTP score, % Identity, and E-value, hits were ranked. From the most likely hit, a reverse BLAST search was conducted: maize genes identified using the approach described above were blasted back to model species to identify orthologous genes, the goal was to confirm that the gene with the highest score was the original BR/GA pathway gene from model species. If the gene identified was not the original gene (used to find maize candidate genes), it was discarded. Finally, each BR/GA gene from the model species was aligned with at least one candidate gene in maize, with a few genes aligned with 2–4 maize candidate genes (which are likely due to gene duplications in maize). Finally, we compared the GA candidate genes found in this study with GA candidate genes published with another method (Song et al., 2011), which used all previously reported genes encoding GA metabolism enzymes in maize and other species as BLAST queries.

### Co-localization of BR and GA Candidate Genes and PHT Associated SNPs

We used a bin size of 1 Mb according to a previous protocol (Yang et al., 2015) around each PHT associated SNP to capture BR/GA candidate genes. Moreover, we used a re-sampling based non-parametric method to test the hypothesis, that the observed number of BR or GA candidate genes co-localized with PHT associated SNP markers using a 1 Mb bin size is not different from randomly occurrence. First, we defined the number of PHT associated SNP markers capturing BR/GA candidate genes using a 1 Mb bin size as “observed value.” Second, the same number of PHT associated SNP markers from GWAS results was randomly sampled (sampling with replacement) from the whole set of SNP markers. In each random sample, all sampled SNP markers used a bin size of 1 Mb to capture BR/GA candidate genes. The number of SNP markers capturing a BR or GA candidate gene was recorded as test statistic. This procedure was repeated for 10,000 times, and the number of test statistics exceeding the observed value was divided by the total number of simulations to compute a *P*-value (Johnson et al., 2011; Yang et al., 2015).

### Path Analysis between BR, GA, PHT, and Heterozygosity

For each PIF, we recorded the BR inhibitor response, GA inhibitor response, PHT performance and the level of heterozygosity (the percentage of heterozygous markers). We used path analysis implemented in IBM SPSS Amos 20 (Arbuckle and Amos, 2006; Jansen et al., 2013) to investigate the direct or indirect correlations between these four variables by fitting proposed path models according to the covariance structure of the underlying data (Jansen et al., 2013). The goal is to explore the hypothesis that heterozygosity is impacting PHT through the regulation of BR and GA level in maize heterozygotes. The correlations between each pair of these four variables are used to calculate total, direct, and indirect effects between the observed variables. We started with a proposed model with the hypothesis that the level of heterozygosity indirectly impacts PHT through the regulation of BR and GA instead of a direct correlation. In other words, there are no other factors connecting heterozygosity and PHT except for BR and GA. Based on the data structure, path analysis suggests to add new links or to delete old links between variables. The final best fit was defined by a stringent criterion with Chi-squared *P*-value > 0.05, root mean square error of approximation (RMSEA) < 0.05, comparative fit index (CFI) > 0.95, goodness of fit index (GFI) > 0.8, adjusted goodness of fit index (AGFI) > 0.9, normed fit index (NFI) > 0.9, relative fit index (RFI) > 0.9, incremental fit index (IFI) > 0.9. If more than one model fit all criteria, then selection was based on a minimum Akaike’s information criterion (AIC) value (Thompson, 1988; Jansen et al., 2013).

## Results

### Phenotypic Characterization

**FIGURE 1:**
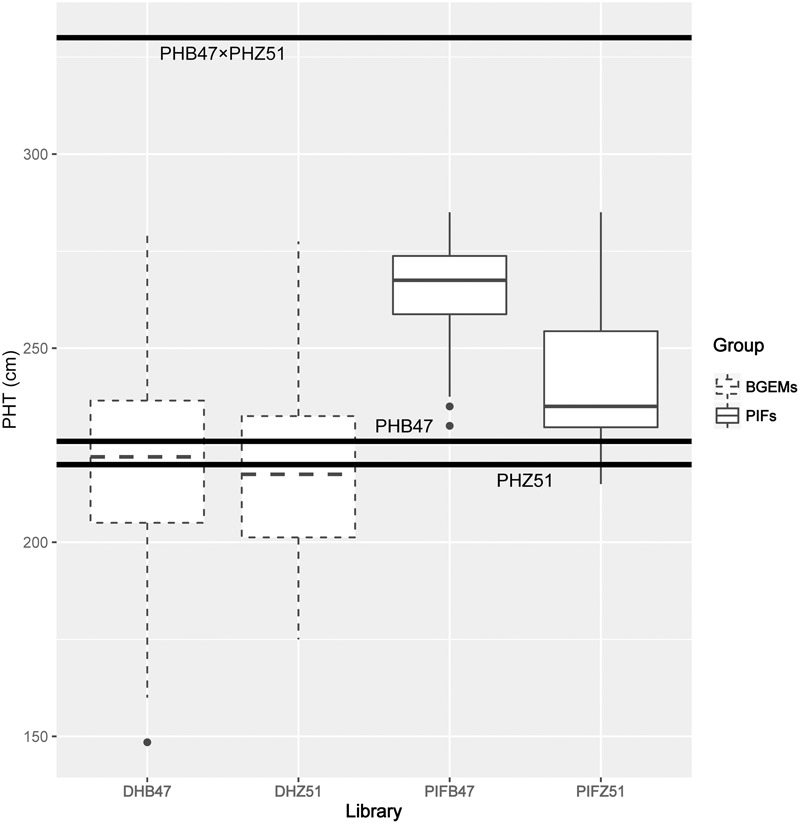
Box plots of PHT performance of the BGEM lines and PIFs. The box in the middle between the first (Q1) and third quartile (Q3) represents 50% values. The lines within in each box indicate the median value for each group. The three bold horizontal lines represent the F_1_ hybrid of PHB47 × PHZ51, PHB47, and PHZ51, respectively.

All statements about statistical inferences are based on a type I error rate (or family-wise error rate) of 5% unless *P*-values are specifically given in the Section “Results.” As described in the Section “Materials and Methods,” two libraries of phenotypic-selected introgression families (PIFs): PIFB47 (PHB47 as recurrent parent) and PIFZ51 (PHZ51 as recurrent parent), and two doubled haploid libraries (DHB47 and DHZ51, without any phenotype selection) were compared with their recurrent parent. Most PIFs were significantly taller than their recurrent parent (**Figure 1**) and showed consistent PHT performance (heritability: h^2^ = 0.81) across 3 years. The percentages of PIFs significantly taller than their recurrent parent in each year were: 91.6% (2013), 92.5% (2014), 92.0% (2015) for PIFB47; and 85.7% (2013), 85.5% (2014), and 90.0% (2015) for PIFZ51. Even for the shortest individual within each PIF (SPHT), 91.2 and 59.4% of these individuals were taller than recurrent parent within PIFB47 or PIFZ51, respectively. In contrast, the proportion of taller BGEM lines (DH lines from DHB47 and DHZ51: see Materials and Methods) compared with their recurrent parent was only 23% for DHZ51 and 18% for DHB47. PIFB47 was on average 23 cm taller than PIFZ51, and PHB47 was on average 8 cm taller than PHZ51. For the BGEM lines, DHB47 was on average 2 cm taller than DHZ51. None of the PIFs were significantly taller than the F_1_ hybrid of PHB47 × PHZ51.

All PIFs flowered within 3 days compared to the respective recurrent parent for both days to tasseling (DDT) and DTS. The average ASI was 0.3 and 0.6 days for PIFB47 and PIFZ51, respectively, and thus similar to PHB47 (0.2 days) and PHZ51 (0.4 days). Differences of DDT and DTS between BGEM lines and recurrent parent ranged from 0–13 days in DHB47 and 0–15 days in DHZ51. The F_1_ hybrid of PHB47 × PHZ51 flowered 4 or 6 days earlier than PHB47 and PHZ51, respectively. Selection for PHT affected other agronomic traits including ear height (EHT), number of nodes (NNode), and tassel length (TL) but not leaf angle (LA). EHT, NNode, and TL increased along with an increased PHT (**Table 1**) and LA is not associated with other traits. Summary statistics for other traits are listed in **Supplementary Table S3**.

**Table 1:** Phenotypic correlations between different agronomic traits in PIFB47 and PIFZ51.

*PIFZ51*	PHT	EHT	TL	NNode	LA
PHT	–	0.70^∗∗^	0.36^∗∗^	0.31^∗∗^	-0.05
EHT		–	0.26^∗∗^	0.28^∗∗^	0.19^∗^
TL			–	-0.01	0.04
NNode				–	-0.17^∗^
LA					–

***PIFB47***	**PHT**	**EHT**	**TL**	**NNode**	**LA**

PHT	–	0.73^∗∗^	0.38^∗∗^	0.31^∗∗^	0.1
EHT		–	0.28^∗∗^	0.25^∗∗^	0.15
TL			–	0.05	0.09
NNode				–	0.04
LA					–

*^∗^α = 0.05 and ^∗∗^α = 0.01, respectively. PHT, EHT, TL, NNode and LA represents for plant height, ear height, tassle length, number of nodes, and leaf angle, respectively.*

### Genotypic Characterization

We defined donor genome proportion (DGP) of PIFs as 50% of heterozygosity (percentage of heterozygous markers) in this study, as for each heterozygous locus, only one allele is from the donor parent. On average, PIFs had a higher DGP than expected by chance (**Table 2**). For example, the expected DGP without selection is on average 0.78% for BC_6_ individuals, but the observed DGP for BC_6_ PIFs was 17.2 and 6.2% in PIFB47 and PIFZ51, respectively. On average, PIFB47 contained 12.9% higher DGP compared to PIFZ51. In contrast, BGEM lines showed a slightly reduced average DGP of 22.3% in DHB47 and 23.8% in DHZ51, compared with the expected 25% of BC_1_-DH. DGP and PHT are significantly correlated for both PIFB47 (*r* = 0.72) and PIFZ51 (*r* = 0.70). However, there was no significant linear relationship between DGP and PHT for BGEM lines.

**Table 2:** Genetic characterization of PIFB47 and PIFZ51.

Generation	Expected DGP (%)	Observed DGP (%) of PIFB47	Observed DGP (%) of PIFZ51
BC_3_	6.25	17.3	4.1
BC_4_	3.13	16.7	2.7
BC_5_	1.56	17.1	4.9
BC_6_	0.78	17.2	6.2

*DGP represents for donor genome proportion.*

### Hormone Inhibitor Responses of PIFs and BGEM Lines

**FIGURE 2:**
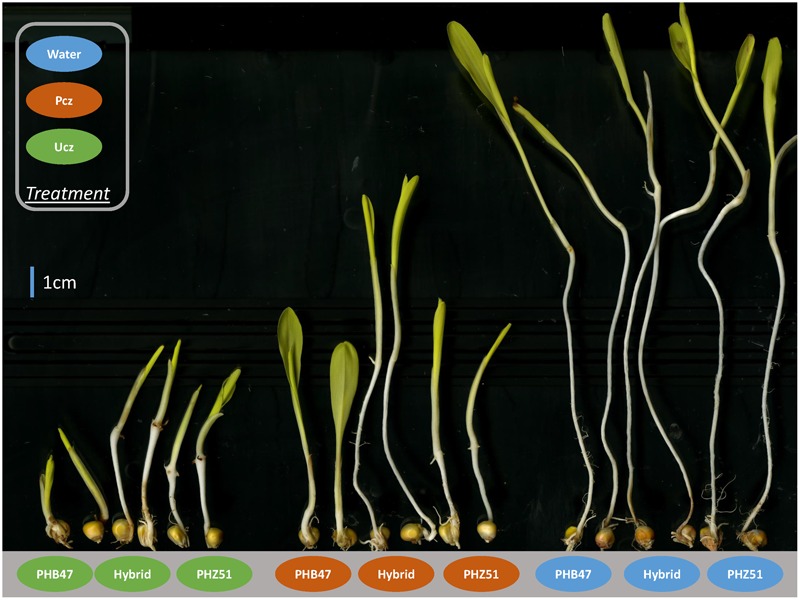
Hormone inhibitor responses of PHB47, PHZ51 and their F_1_ hybrid of PHB47 × PHZ51. From left to right, PHB47, PHZ51, F_1_ hybrid of PHB47 × PHZ51 were treated with gibberellin inhibitor Ucz (green), brassinosteroid inhibitor Pcz (orange), and Water (blue), respectively.

**FIGURE 3:**
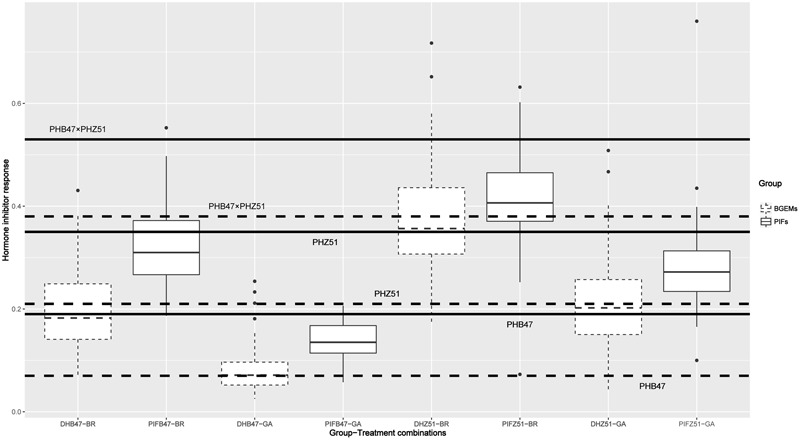
Hormone inhibitor responses of BGEM lines and PIFs. X-axis: groups from left to right represent different combinations of library and hormone inhibitor treatments. For example, DHB47-BR represents the DHB47 library treated with BR inhibitor. Y-axis reflects the hormone inhibitor response which is calculated as (mesocotyl length with hormone inhibitor treatment)/(mesocotyl length with water treatment). The box in the middle between the first (Q1) and third quartile (Q3) represents 50% values. The lines within in each box indicate the median value for each group. Solid and dashed lines indicate BR and GA treatment, respectively. The six horizontal lines from top to bottom indicate F_1_ (hybrid of PHB47 × PHZ51) BR inhibitor response, F_1_ GA inhibitor response, PHZ51 BR inhibitor response, PHZ51 GA inhibitor response, PHB47 BR inhibitor response, PHB47 GA inhibitor response, respectively.

We applied BR and GA inhibitors to PIFB47, PIFZ51, DHB47, and DHZ51 to calculate the BR and GA inhibitor responses. The BR and GA inhibitor Pcz and Ucz greatly reduced mesocotyl length compared to water treatment (**Figure 2**). Heritabilities of BR inhibitor response (defined as MP/MW; see Materials and Methods) and GA inhibitor response (MU/MW) were 0.74 and 0.80, respectively. Ucz showed stronger effects than Pcz for reducing the mesocotyl length for both PIFs and BGEM lines (**Figure 3**). PHZ51 showed stronger BR and GA inhibitor response compared to PHB47. MP/MW was 0.19 and 0.35 for PHB47 and PHZ51, and MU/MW was 0.07 and 0.20 for PHB47 and PHZ51, respectively. 69% (PIFB47) and 45% (PIFZ51) of PIFs had increased BR inhibitor response (more tolerant to the BR inhibitor, in other words with an elevated BR level or signaling) compared to their respective recurrent parent. GA inbititor response was 68% (PIFB47) and 38% (PIFZ51) increased for PIFs, compared to their respective recurrent parent. In contrast, only 6–13% of BGEM lines showed significant increased BR/GA inhibitor response compared to their recurrent parent, and the BR and GA inhibitor responses of the recurrent parents were always close to the median of DHB47 and DHZ51 (without phenotype selection) (**Figure 3**). The F_1_ hybrid of PHB47 × PHZ51 showed significantly higher hormone inhibitor response for both BR and GA than most of the PIFs. Only one PIF from PIFZ51 showed higher BR inhibitor response than the hybrid of PHB47 × PHZ51 (**Figure 3**).

### BR and GA Control of PHT

**FIGURE 4:**
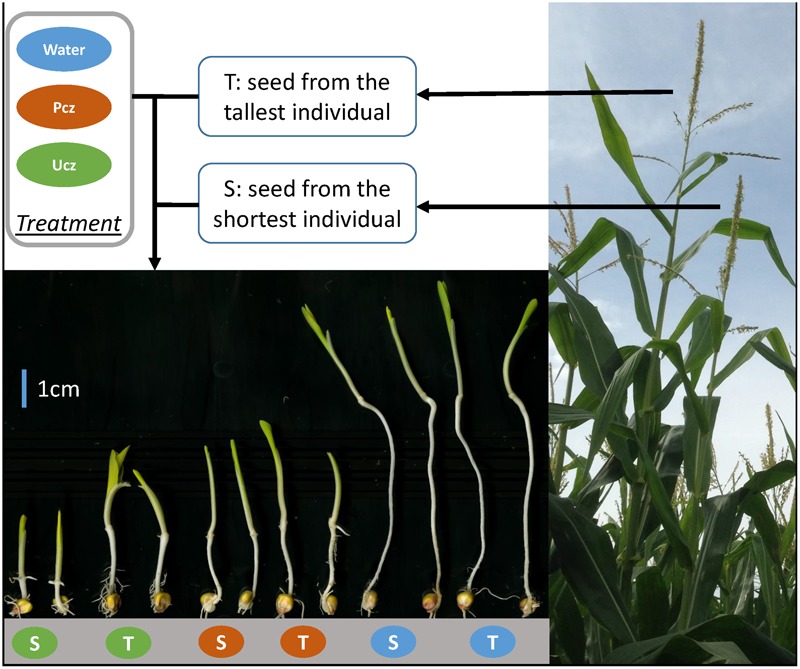
Hormone inhibitor response of seed from tallest and shortest individuals of PIF113. Green, orange, and blue represents for gibberellin inhibitor Ucz, brassinosteroid inhibitor Pcz, and water treatment, respectively.

Within both PIFB47 and PIFZ51: BR and GA inhibitor responses, and PHT were significantly correlated (**Table 3**). PIFs with stronger BR or GA inhibitor response were taller in the field. In other words, the PIFs which were more tolerant to BR and GA inhibitors were also taller. In contrast, no significant correlation was found between BR inhibitor response and PHT or between GA inhibitor response and PHT for BGEM lines. This indicates that the relationship between BR, GA, and PHT was different in heterozygotes verses inbred lines. When assessed between the tallest individual and the shortest individual within PIFs, seed harvested from the tallest individual showed stronger hormone inhibitor response (either BR or GA or both) compared with the seed harvested from the shortest individual (**Supplementary Table S4**). For example, seed from the tallest individual was more tolerant to GA inhibitor compared to the seed from the shortest one of PIF113 (*P* = 0.0003) (**Figure 4**). These PIFs contained a high level of heterozygosity (%) with on average 40.4% (PIFB47) and 17.8% (PIFZ51).

**Table 3:** Correlations between BR/GA inhibitor response, PHT, and heterozygosity for PIFs and BGEM lines.

PIFB47	PHT	BR	GA	Heterozygosity
PHT	–	0.46^∗∗∗^	0.59^∗∗∗^	0.72^∗∗∗^
BR		–	0.52^∗∗∗^	0.34^∗∗^
GA			–	0.53^∗∗∗^
Heterozygosity				–
**PIFZ51**	**PHT**	**BR**	**GA**	**Heterozygosity**
PHT	–	0.51^∗∗∗^	0.58^∗∗∗^	0.70^∗∗∗^
BR		–	0.54^∗∗∗^	0.32^∗∗^
GA			–	0.41^∗∗∗^
Heterozygosity				–
**DHB47**	**PHT**	**BR**	**GA**	–
PHT	–	-0.12	-0.07	–
BR		–	0.68^∗∗∗^	–
GA			–	–
**DHZ51**	**PHT**	**BR**	**GA**	–
PHT	–	-0.08	-0.04	–
BR		–	0.49^∗∗∗^	–
GA			–	–

*^∗∗^α = 0.01, ^∗∗∗^α = 0.001, respectively; BR and GA represents for brassinosteroid and gibberellin inhibitor response, respectively; PHT represents for plant height.*

**FIGURE 5:**
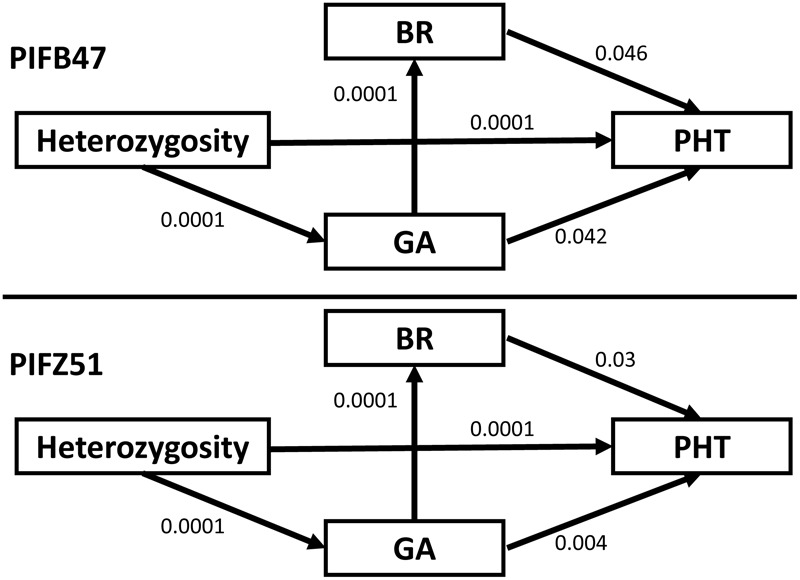
The final best model from path analysis for PIFB47 and PIFZ51. Heterozygosity represents for the proportion of heterozygous markers for PIFs. BR and GA represents for brassinosteroid and gibberellin inhibitor response, respectively. PHT represents for plant height. The numbers represent for *P*-values of direct relationships between each two variables (without confounding effects).

Path analysis was used to connect the level of heterozygosity, BR inhibitor response, GA inhibitor response, and PHT separately within both PIFB47 and PIFZ51. Starting from the proposed model (see Materials and Methods), new links were added and old links were deleted based on the correlation structure. Path analysis for PIFB47 and PIFZ51 arrived at the same final best model, for which the direct correlation between heterozygosity and BR was deleted, and new links between BR and GA, and between heterozygosity and PHT was added (**Figure 5**) compared to the original proposed model. This was the only model fitting all criteria and it was associated with the lowest AIC value. According to the final model a high level of heterozygosity has a direct positive effect on PHT and two indirect paths to impact PHT: (i) a higher level of heterozygosity increases the GA level (synthesis or signaling), and increased GA promotes PHT; (ii) increased GA causes an increased BR level/signaling, which then promotes PHT.

### Genome-Wide Association Analysis

**FIGURE 6:**
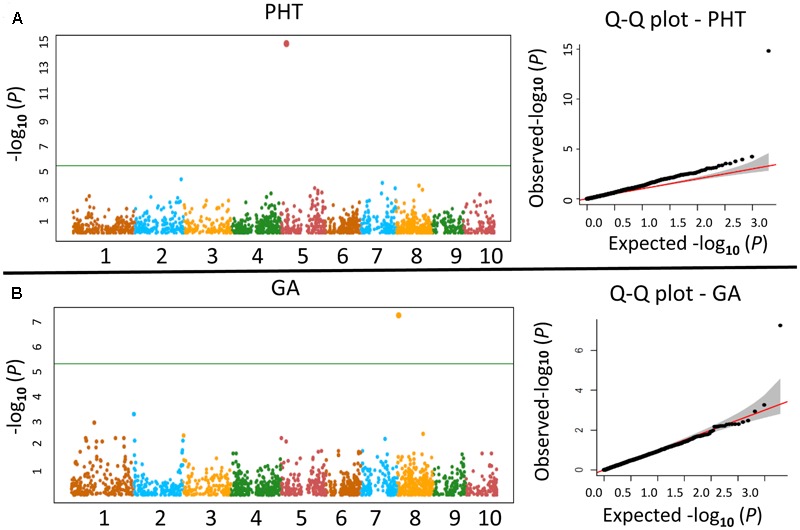
Manhattan plot and QQ-plot of the FarmCPU results for plant height (PHT) and gibberellin inhibitor response (GA). **(A)** X-axis represents for the 10 chromosomes in maize and Y-axis represents for negative log_10_-transformed *P*-values. **(B)** X and Y axis represents for the expected and observed negative log_10_-transformed *P*-values for FarmCPU model, respectively.

Three sub-populations were obtained for joint analysis of PIFB47 and PIFZ51, with two subpopulations containing PHB47 and PHZ51, respectively (with limited donor introgression), and a mixed subpopulation containing a large (∼30%) DGP. With three GWAS models MLM, GLM+Q, FarmCPU for balancing false positives and false negatives (see Materials and Methods), we found that none of the SNP markers was found to be significantly associated with BR/GA inhibitor responses, PHT and other agronomic traits with a MLM (Q+K) model, which corresponds to previous studies that MLM is a very stringent model. With the FarmCPU method [Q+K model, but with controlled confounding effect between covariates (Q, K) and testing markers], we found one SNP SYN38535 on Chromosome 5 (282,014,45 bp; *P*-value 1.39 × 10^-15^) significantly associated with PHT (**Figure 6**) and one SNP PZE-108005623 on Chromosome 8 (567,899,5 bp; *P*-value 5.45 × 10^-8^) significantly associated with GA inhibitor response (**Figure 6**). QQ-plot showed that population structure and kinship controlled false positives effectively (**Figure 6**). The number of associated loci was limited either due to the model stringency or limited number of markers, but detected loci has a high probability to be true positive. With a GLM+Q model, 73 SNPs were significantly associated with PHT (**Supplementary Table S5**) including SYN38535 from FarmCPU, and 22 of them overlapping with the 40 published PHT QTL hot spots. This corresponds to previous studies that GLM+Q model is less stringent compared to MLM and FarmCPU. Of these 73 SNPs, 41 were located within genes and these gene functions were summarized in **Supplementary Table S6** according to the function of their orthologes in rice and Arabidopsis. Among these genes, the four genes *GRMZM2G348452, GRMZM2G082191, GRMZM2G100423*, and *GRMZM2G025171* are promising for further investigation, as the orthologes of these four genes – cytokinin oxidase, receptor-like protein kinase, cytochrome P450 and ATP synthase in Arabidopsis and rice are functioning in PHT control (Li et al., 2002; Komorisono et al., 2005; Zhu et al., 2006; Gao et al., 2014).

For GA and BR inhibitor response, we found five, and two significant SNPs with GLM+Q method, respectively (**Supplementary Table S5**), with one overlapping SNP marker SYN19928 on Chromosome 8 (128,133,446 bp) associated with both BR (*P*-value 3.62 × 10^-5^) and GA (*P*-value 1.66 × 10^-5^) inhibitor response. This SNP marker was found to be the closest linked marker in our dataset to a BR signaling pathway candidate gene *ZmBSU1* with a 200 kb physical distance. For the other four SNPs associated with GA inhibitor response, three were close to GA candidate genes: PZE-108005623 (overlapped with FarmCPU method) and PZA00058.6 (Chromosome 8; 602,357,7 bp) are clustered and 2 Mb away from GA signaling candidate gene *ZmRBX1A*. Marker PZE-108070783 (Chromosome 8; 123,786,918 bp) is 3 Mb away from GA signaling candidate gene *ZmCUL1*. These three candidate genes *ZmBSU1, ZmRBX1A*, and *ZmCUL1* are expressed at seedling stage with the same absolute expression value of 3475.53 (Sekhon et al., 2011). For the other traits, four markers were associated with EHT, one with NNode, and one with LA (**Supplementary Table S5**). None of these markers were close to any BR or GA candidate genes.

### Co-localization of BR and GA Candidate Genes with PHT Associated SNP Markers

We systematically used ortholog information from Arabidopsis and rice to find all GA and BR candidate genes in maize. Compared with a different method, which used all previously reported genes encoding GA metabolism enzymes in maize and other species as BLAST queries to find GA candidate genes, all the GA candidate genes found in this study matched with their results (Song et al., 2011). With 1 Mb bin size, seven PHT associated SNP markers co-localized with GA candidate genes (**Table 4**). Except for marker PZA02388.01 and PZE-108073190, which are the third and second closest SNP markers to the candidate gene, all the other SNP markers are the closest SNP markers included in our marker dataset to the candidate gene. The probability of finding seven PHT associated SNP markers co-localized with GA candidate genes by chance with a 1 Mb bin size within our marker dataset is *P* = 0.041 based on a non-parametric resampling method. We found 2 BR candidate genes co-localized with PHT associated SNP markers with 1 Mb bin size (**Table 4**), and both of them belongs to a BR catabolic gene family BAS1.

**Table 4:** Co-localization of gibberellin (GA) candidate genes and PHT loci.

Marker	Chr	Position (bp)	Distance (Mb) to candidate gene	Candidate gene	Pathway	Candidate gene ID
PZE-101087901	1	792,367,10	0.47	*ZmCPS3*	GA biosynthesis	*GRMZM2G044481*
PZE-103056753	3	695,588,88	0.39	*ZmDELLAs-1*	GA signaling	*GRMZM2G001426*
PZE-106070979	6	125,452,957	0.43	*ZmGA2ox7*	GA biosynthesis	*GRMZM2G078798*
PZA02388.1	8	169,137	0.77	*ZmPIF3-2*	GA signaling	*GRMZM2G387528*
PZE-108073190	8	127,625,290	0.75	*ZmCUL1-1*	GA signaling	GRMZM2G166089
SYN13209	8	164063270	0.87	*ZmGA20ox4*	GA biosynthesis	GRMZM2G049418
PZE-109092637	9	139661789	0.36	*ZmKAO1*	GA biosynthesis	GRMZM2G089803
SYN29112	4	231412099	0.6	*ZmBAS1*	BR catabolic	GRMZM2G107322
PZE-109049656	9	86397696	0.18	*ZmBAS1*	BR catabolic	GRMZM2G107322

## Discussion

### Impact of Heterozygosity on Increased PHT in PIFs

Phenotypic selection for PHT and flowering time was successful in this study. Most PIFs (∼90%) were significantly taller than their respective recurrent parent (**Figure 1**), while flowering undistinguishable from their recurrent parents. Selection for PHT resulted in high levels of heterozygosity (**Table 2**). The percentage of heterozygous markers for PIFB47 and PIFZ51 after four to six generations of backcrossing was on average 34 and 9%, respectively. Percentage of heterozygosity was correlated with PHT in both PIFB47 and PIFZ51 (*r* ∼ 0.7). In contrast, only about 20% of BGEM lines were significantly taller than their recurrent parent, and there was no significant correlation between DGP and PHT. Without selection, the observed DGP is around 23%, close to the expected 25% for BC_1_ derived doubled haploid lines.

Stronger selection was observed in PIFB47. PIFB47 was on average 23 cm taller than PIFZ51, and this difference was larger than the difference between PHB47 and PHZ51 (8 cm). Moreover, PIFB47 contained on average 25% more heterozygous markers than PIFZ51. In comparison, DHB47 was on average 2 cm taller than DHZ51, and both of them had an observed DGP around 23%. In addition, we compared a subset of PIFs from PIFB47 and PIFZ51, which were derived from the same set of donor parents backcrossed with both PHB47 and PHZ51. On average, these PIFs were 30 cm taller for PIFB47 compared to PIFZ51. The stiff stalk group (including PHB47) is more distantly related to tropical germplasm than the non-stiff stalk group (including PHZ51) (Liu et al., 2003). Thus, the chance of heterotic effects increases for crosses between PHB47 and tropical germplasm. Previous studies for U.S. maize germplasm showed that panmictic midparent heterosis (PMPH) linearly increased with increasing genetic distance (Moll et al., 1962), and PMPH also increased with increasing genetic distances among tropical and U.S. germplasm, unless affected by maladaptation problems (Moll et al., 1965). An increased divergence between two genotypes of a heterotic pattern increases the probability to select for complementary favorable alleles at different loci (Reif et al., 2005), which is consistent with our findings of accumulating more exotic regions at heterozygous state in PHB47 background.

### BR and GA Inhibitor Responses Are Correlated with PHT for PIFs

Brassinosteroid and GA are two classes of plant hormones that are regarded as major pathways controlling PHT (Wang and Li, 2008). The effect of BR and GA on PHT is manifested primarily through enhanced internode elongation resulting from both increased cell elongation and cell division (Zhang et al., 2007; Fernandez et al., 2009). Previous studies focused on two aspects to connect BR, GA, and PHT in maize from a genetic perspective: (1) identification of genes with profound effect on PHT leading to dwarf mutants, caused by defects in BR or GA synthesis or signaling pathway genes (Peng et al., 1999; Wang and Li, 2008; Fernandez et al., 2009; Hartwig et al., 2011; Makarevitch et al., 2012); (2) identification of PHT QTL, co-localized with BR and GA candidate genes (Fernandez et al., 2009; Weng et al., 2011; Teng et al., 2013; Peiffer et al., 2014). In this study, we found seven co-localized GA candidate genes with PHT associated SNP markers using a 1 Mb bin size, and this observed frequency of co-localization is significantly different from random co-segregation. In addition, two BR candidate genes were found to be co-localized with two PHT associated SNPs. With a diverse panel of 7000 accessions, Yang et al. (2015) successfully discovered co-localizations with 1 Mb bin size. Considering the limited number of backcross generations for generating PIFs, 1 Mb is a reasonable cutoff to serve as a linkage block in this study. In maize, there are around 32,000 genes predicted, with a 2700 Mb genome size of maize (Schnable et al., 2009). On average, there are around 32000/2700 = 12 genes within a 1 Mb region. Our assumption is, that BR and GA candidate genes are promising candidates for PHT control. In sorghum, two recent studies have used the Sequenom (SQNM) MassARRAY iPLEX platform (Gabriel et al., 2009) to develop molecular markers from GA and BR candidate genes (Perez et al., 2014; Zhao et al., 2016) for association studies, this method is promising to be applied in maize populations with low linkage disequilibrium but do not add more information for populations with extended linkage blocks such as in PIFs.

Previous studies used Pcz and Ucz as BR and GA inhibitors to phenocopy the effect of BR and GA biosynthetic gene mutations (Hartwig et al., 2012). With Pcz and Ucz treatment, mesocotyl length of maize seedlings is significantly reduced, and genotypes with higher BR and GA biosynthesis or signaling show increased tolerance to corresponding inhibitors. In this study, we applied Pcz and Ucz to compare the BR and GA inhibitor responses among PIFs and BGEM lines. We found that the pattern of hormone inhibitor responses was very similar to the pattern of PHT performance (**Figures 1, 3**), as PIFs are on average more tolerant to both BR and GA inhibitors compared to their respective recurrent parents and BGEM lines, and PIFs are also much taller compared to respective recurrent parents and BGEM lines. In addition, when compared between different PIFs – taller PIFs are associated with stronger BR and GA inhibitor response within both PIFB47 and PIFZ51 (**Table 3**). Even within the same PIF, there were significant differences for hormone inhibitor response between offspring from the tallest and the shortest plants. In this study, the only selection applied was for PHT, not for hormone inhibitor response. This indicates that (1) “stronger” hormone pathway genes were directly selected for by selecting for increased PHT, or (2) there was a common pathway contributing to both taller PHT and increased hormone inhibitor response for PIFs.

### Crosstalk between BR and GA

We found significant correlations between BR and GA inhibitor responses for both the PIFs and BGEM lines (**Table 3**). PIFs and BGEM lines that are more tolerant to one of these two hormone inhibitors also tend to show tolerance to the other hormone inhibitor. Pathway analysis suggested that there was a direct crosstalk between the BR and GA pathways, supported by recent studies in Arabidopsis and rice (Bai et al., 2012; Gallego-Bartolomé et al., 2012; Li et al., 2012; Tong et al., 2014; Hofmann, 2015; Unterholzner et al., 2015). In addition, we found the same SNP, co-localized with a BR candidate gene *ZmBSU1*, to be associated with both BR and GA. This indicates that BSU1 may have a function in the interaction between BR and GA. A previous study showed that BZR1, a transcription factor activated by BR signaling and a DELLA protein, which inhibits the GA signaling pathway, are the key genes mediating crosstalk between BR and GA signaling pathways (Li et al., 2002; Bai et al., 2012). Since BSU1 is activating BZR1 activity (Ye et al., 2011), it explains detection of BSU1 as a modulator.

### Prediction of Heterosis in PHT by Early Monitoring of BR and GA Levels

Heterosis and inbreeding depression are considered two sides of the same coin (Mather and Jinks, 2013) and both of them are defined and quantified in relation to a reference population. Any effect of inbreeding in a population will increase heterosis by the same amount (Miranda and Brasil, 1997). Heterosis is clearly related to heterozygosity, but it has long been debated how heterozygosity results in heterosis (Li et al., 2001). Inbreeding depression is caused by increased homozygosity of individuals (Charlesworth and Willis, 2009), as increased levels of homozygosity accumulate detrimental recessive mutations and reduce heterozygote advantages. Positive correlations between trait expression and level of heterozygosity are recognized as suggestive evidence for heterosis and inbreeding depression, and inbreeding coefficients estimated using homozygous SNPs was found to correlate well with pedigree inbreeding coefficient to infer inbreeding depression (Kim et al., 2015). In this study, we found significant positive correlations between PHT and the level of marker heterozygosity (*r* ∼ 0.7). Path analysis indicates that the level of heterozygosity is directly and positively correlated with GA levels (*r* ∼ 0.5), and seedling stage BR/GA inhibitor responses were positively correlated with field PHT for PIFs (*r* ∼ 0.5), but not BGEM lines (*r* ∼ 0.1). We were not able to directly correlate BR/GA with heterosis in PHT, as we only have recurrent parent and backcross progeny information (exotic donor parents are segregating accessions without available seed source and characterization). However, we were able to calculate the level of homozygosity, and correlate the level of homozygosity with PHT. Increased inbreeding significantly reduced PHT, with correlation (between the level of homozygosity and PHT) around 0.7. As the level of homozygosity is closely correlated with inbreeding depression (Kim et al., 2015), and inbreeding depression is the flip side of heterosis, we conclude that BR and GA promote heterosis for PHT (α = 0.05) in this study.

Previous studies have shown that GA levels are correlated with seedling heterosis: (1) hybrids were associated with higher GA levels than parental inbred lines in maize (Rood et al., 1988, 1992), sorghum (Rood et al., 1992), poplar (Bate et al., 1988) and rice (Ma et al., 2011), and when heterosis was not displayed due to unfavorable environmental conditions, the hybrid contained equal levels of endogenous GA-like substances (Rood et al., 1985) as the inbred parents; (2) maize inbred lines were more responsive to external application of GAs compared to hybrid progeny, suggesting a deficiency of endogenous GAs in inbred lines (Rood et al., 1988); (3) GA biosynthesis and positive signaling components were up-regulated in hybrids, whereas genes deactivating bioactive GAs, and negative GA signaling components were down-regulated, which together increase seedling heterosis (Ma et al., 2011). In addition to seedling heterosis, GA content was also found to be correlated with heterosis in PHT: it was reported that increased elongation of the uppermost internode contributed most to heterosis for PHT in wheat hybrids (Zhang et al., 2007). Examination of GA levels and activities in the uppermost internode tissue of wheat hybrids revealed, that (1) genes promoting GA biosynthesis were up-regulated and GA deactivating genes were down-regulated, which resulted in higher level of GA in hybrids; (2) upregulation of GA receptors GID1 (for GA INSENSITIVE DWARF1) and positive regulator GAMYB, and down-regulation of negative component GAI resulted in enhanced GA sensitivity; (3) GA promoted the expression of expansion genes such as gibberellins induced proteins (GIPs) and endoxyloglucan transferase (XET), which promoted cell division and cell elongation, finally contributed to increased internode elongation and heterosis in PHT. After 4 days of germinating rice seed, the content of GA_4_ started to be significantly higher in hybrids compared to their inbred parents (Ma et al., 2011). At both seedling and adult stages, GA levels were increased in hybrids. This explains why the seedling stage GA level was correlated with heterosis in PHT for PIFs. There are only few correlation studies between BR and heterosis available, although BRs function in both cell division and elongation in model species rice and Arabidopsis (Szekeres et al., 1996; Hu et al., 2000; Zhiponova et al., 2013; Tong et al., 2014). Despite BRs’ broad effects on the physiological and developmental processes of plants, they were not widely recognized as plant hormones until the mid-1990s (Clouse, 1996; Divi and Krishna, 2009). We observed that the BR level was increased for PIFs with higher levels of heterozygosity, and path analysis indicated that this was due to an indirect effect from the crosstalk with the GA pathway. Nonetheless, BR was shown to directly increase PHT. BR and GA coordinately promote plant growth and development by jointly regulating the expression of specific groups of genes (Yang et al., 2004). Heterosis for PHT is also affected by other factors. For example, we did not measure the auxin response due to the complexity of the auxin biosynthesis pathway (Zhao, 2010), but polar auxin transport has been shown to affect PHT (Wang and Li, 2008).

Both DNA markers, transcripts, and metabolites have been evaluated for prediction of heterosis with various approaches (Andorf et al., 2010; Maenhout et al., 2010; Steinfath et al., 2010). Molecular markers associated with genomic regions contributing to heterosis can be identified with linkage or association mapping methods, and used in a linear regression approach to predict heterosis (Vuylsteke et al., 2000; Basunanda et al., 2010; Meyer et al., 2010; Tang et al., 2010). The advantage of transcriptome based prediction of heterosis over DNA markers is that transcript abundancies are resulted from the integration of the whole genome information, including DNA methylation level and histone modification status, thus the prediction accuracy is higher (Scholten and Thiemann, 2013). For metabolism based prediction of heterosis, metabolic markers were used for the prediction of biomass heterosis in Arabidopsis (Andorf et al., 2010), as metabolite levels are the result of more genes than those represented by genetic markers. Here, we used plant hormone inhibitors to assess the BR and GA level in different maize genotypes, and this information is significantly correlated with heterosis in PHT. With both seedling stage BR and GA inhibitor responses incorporated into a linear model, the prediction accuracies (r) are 0.62 in PIFB47 and 0.63 in PIFZ51. Our results indicate the possibility to use seedling stage hormone inhibitor response to predict heterosis for PHT in maize breeding projects, especially for biomass maize production. However, it needs to be noted that our prediction is based on the heterozygotes (PIFs) *per se*, instead of using their parental information. In other words, previous studies used molecular data from inbred parents to predict heterosis, whereas our prediction was from the same genotypes. To further investigate the usage of hormone based prediction, we measured 201 hybrids for PHT across four replications, which were derived from BGEM lines (DHB47) × PHZ51, BGEM lines (DHZ51) × PHB47, and PHB47 × PHZ51. We calculated the Mid-parent heterosis as we measured PHT for F_1_, BGEM lines, PHB47 and PHZ51. We used parental BR and GA inhibitor response to predict Mid-parent heterosis, with data available in **Supplementary Table S7**. Additional phenotypic and genotypic data supporting this research is available in **Supplementary Tables S8–S10**. Neither BR nor GA inhibitor responses of the inbred parents were associated with heterosis in PHT, with correlations less than 0.1. Thus, our hormone based prediction method can only be used to predict adult performance based on seedling information of the same genotype, instead of using inbred parental information to predict heterosis in hybrids.

## Conclusion

In this study, we used phenotypic selection to improve PHT and broadened the genetic variation of two maize heterotic groups (Stiff Stalk and Non-Stiff Stalk) by adapting multiple tropical and subtropical accessions into these two genetic pools. We found that phenotypic selection of PHT was genotype dependent, and stronger selection was observed when crosses were made between Stiff Stalk and tropical germplasm. Our main founding is that, for heterozygotes, GA activities were elevated with an increased level of heterozygosity. Increased GA promotes BR and they together lead to increased heterosis in maize PHT. If this can be generalized for other populations of hybrids (in addition to the crosses between temperate lines and tropical accessions), early stage monitoring of plant hormones is promising for predicting PHT for maize heterozygotes without growing all seed in the field.

## Author Contributions

SH and TL conceived the study, designed the experiments, discussed the results and finalized the manuscript; CW identified the candidate genes in BR and GA pathway; DS collected genotype and phenotype data for doubled haploids as a comparison; AL, PL helped with statistical analysis. YY established protocol for measuring BR/GA inhibitor response. TL, AL, PL, YY, MB edited the manuscript; all authors read and approved the final manuscript.

## Conflict of Interest Statement

The authors declare that the research was conducted in the absence of any commercial or financial relationships that could be construed as a potential conflict of interest.

## Supplementary Material

TABLE S1 Donor parent information and backcross generations for PIFs.

TABLE S2 Maize candidate genes in Brassinosteroid (BR) and Gibberellin (GA) biosynthesis and signaling pathway.

TABLE S3 Summary statistics for other agronomic traits (besides plant height) in PIFB47 and PIFZ51.

TABLE S4 Comparison between the seed from the tallest/shortest individual within one PIF for hormone inhibitor response.

TABLE S5 Markers associated with PHT, EHT, NNode, and LA from association study.

TABLE S6 Plant height (PHT) associated SNP marker annotations and candidate gene functions.

TABLE S7 Raw data for the prediction of Mid-parent heterosis for plant height.

TABLE S8 Raw field phenotype data.

TABLE S9 Raw data for **Table 3**.

TABLE S10 Raw genotypic data.
