# Supplementary material for: Gibberellins Promote Brassinosteroids Action and Both Increase Heterosis for Plant Height in Maize (Zea mays L.)
Source: Front Plant Sci. 2017 Jun 20;8:1039. doi: 10.3389/fpls.2017.01039 (PMC5477294; doi:10.3389/fpls.2017.01039)
Supplement: Supplementary file 11 [file Image_1.PDF]

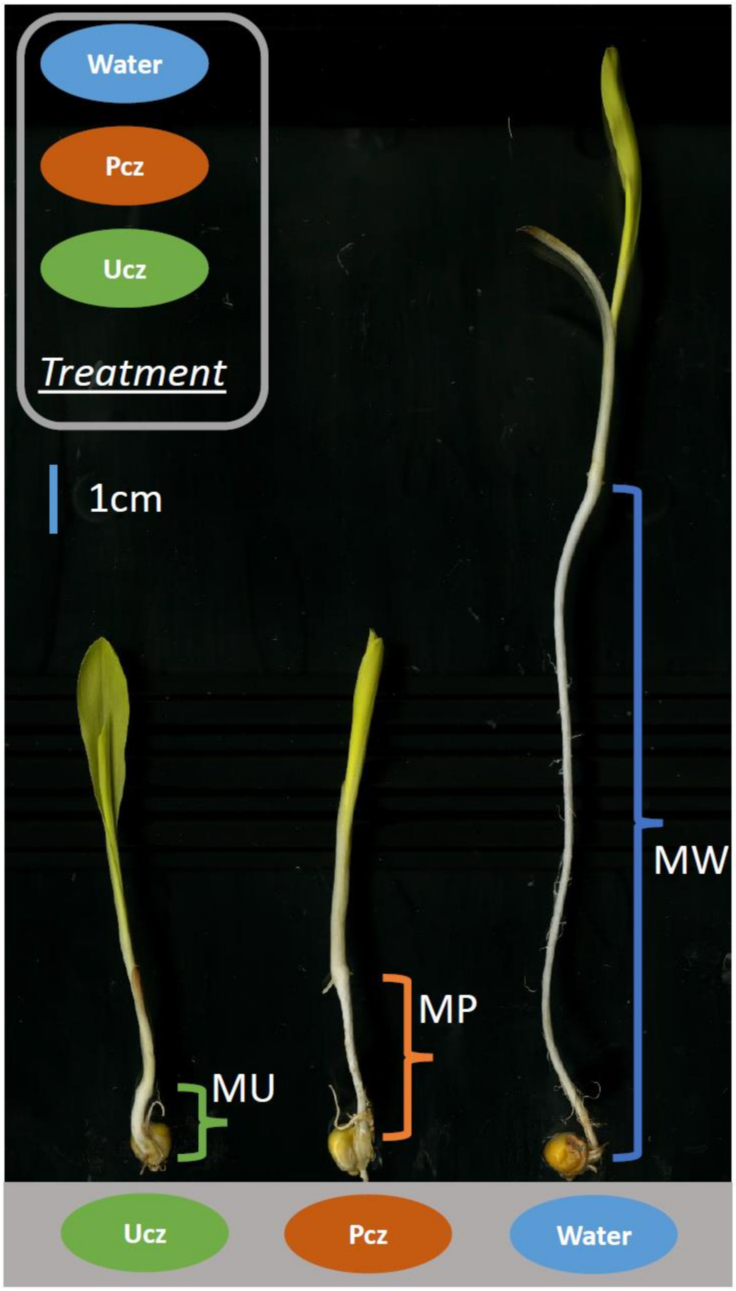

Figure S1: Brassinosteroid (BR) and gibberellin (GA) inhibitor response calculation. Pcz and Ucz represents for BR and GA inhibitor, respectively. MU, MP, MW represents for mesocotyl length of maize seedlings treated with Ucz, Pcz and Water, respectively. BR inhibitor response is defined as MP/MW, and GA inhibitor response is defined as MU/MW.
